# An Antivivisectionist Libel

**Published:** 1904-01

**Authors:** 


A Monthly Review of Medicine and Surgery.

EDITOR:

WILLIAM WARREN POTTER, M. D.

All communications, whether of a literary or business nature, books for review and exchanges should be addressed to the editor: 284 FRANKLIN STREET, BUFFALO, N. Y.

VOL. XLIII.—LIX. JANUARY, 1904. No. 6.

### An Antivivisectionist Libel.

THE antivivisectionists in Great Britain have received a severe reprimand in the law courts,—one it would be well for similar people in our own country to heed. It appears that Mr. Stephen Coleridge,—we omit the rest of his name for the sake of brevity,—a member of the bar, son of the late Lord Chief Justice Coleridge, and an honorary secretary of the Antivivisection Society, had charged Dr. Bayliss, assistant professor of physiology at University College, London, in effect with the ruthless torture and torment of animals upon which he had performed experiments in the course of his instructions to students.

According to the pleadings of counsel Mr. Coleridge prepared a bomb that he fired at the meeting of the Antivivisection Society, at Saint James's Hall, May 1, 1903, and took good care to have a reporter present who published his version in the *Daily News* the next morning. Mr. Coleridge admitted that he had made the statement which the plaintiff complained of, and also his responsibility for what appeared in the *Daily News,* May 2, 1903.

The trial of this action was commenced November 11, 1903, before Lord Chief Justice Alverstone and a special jury. As might be expected the parties to the suit, plaintiff and defendant, were represented by the ablest of counsel, and great general interest attached to the trial which lasted four days. It was specially reported for the *British Medical Journal,* and is printed in its issues for November 14 and 21, 1903, from which we condense this outline. It was shown on the trial that Dr. Bayliss was duly licensed under the law to perform vivisections before his class of students, holding the home secretary's certificate to that effect. The penalty for the first violation of the law by a licensee is $250, and for the second offense is $500. A number of witnesses were examined on both sides, including both plaintiff and defendant, each of whom were questioned in much detail.

The Lord Chief Justice charged the jury that the animal experimented upon must have been under full anesthesia from the beginning to the end of all operative experiments, else the law was violated; that the defendant must establish the fact that such had not been the case in the present instance; otherwise the plaintiff could recover. In other words, the defendant was not entitled to leave the matter in doubt. He further charged it was clearly to be borne in mind that as between comments and criticism on the one hand and allegations of fact on the other, it was one thing to comment upon or criticise with severity, and another to assert that a man had been guilty of particular acts of misconduct. He still further, in concluding his charge, said it was impossible to consider the case a light one and affirmed it was self-evident that Mr. Coleridge was entitled to comment in the strongest possible way, but that a person who undertook to advocate a cause was not entitled to do so by charging other people with criminal offenses unless he was able to substantiate such charges. The Lord Chief Justice's final postulate was that no man had any right to import into the public discussion of public questions slanderous defamation of any other man, unless he was prepared to prove those charges in a court of justice.

We have given the salient features of the summing up by the learned Lord Chief Justice, but have done imperfect justice to his charge. It is in its completed phraseology a clear exposition of the law of libel, according to the statutes of Great Britain. At the conclusion of the charge the jury retired and, after an absence of twenty-five minutes, returned into court with a unanimous verdict for the plaintiff, assessing the damages at £2,000, or about $10,000, together with the costs of the special jury. The verdict was received by the public assembled in the courtroom with demonstrations of satisfaction.

The *British Medical Journal* of November 21, 1903, in commenting editorially on the trial, concludes as follows:

> Here was a test case of the utmost gravity, eye-witnesses, sworn statements, masses of evidences, and a great array of witnesses, to end in absolute defeat, for this one and only reason, that the other side told the truth. We offer to Dr. Bayliss, Professor Starling, Sir Victor Horsley, and all the witnesses on that side, not only our congratulations, but also our heartiest thanks. *Bene meruerunt de republicâ.* They have done their duty by their profession, and have done it well. For their reward, they know that they have taught everybody to doubt the argument and the literature that are the stock-in-trade of these antivivisection societies. The arguments, alas, will go on, and so will the literature. But, henceforth, the sting is gone out of the angry abusive language, and the catchword "torture" will be taken for what it is worth. The recent trial has shown once more that these societies are not to be trusted. The chief of them, the society that held its head highest and had the proudest list of titled supporters, has been put to the test in the person of its chief representative, and the result of that test can hardly be misunderstood.

---

THE epidemic of typhoid fever at Butler, Pa., has assumed startling proportions, and before it is stamped out will undoubtedly become the most formidable of its kind that the world has yet seen. In a population, aggregating about 15,000, there were reported up to December 14, 1,247 cases, with 51 deaths. Dr. George A. Soper, a sanitary expert employed to investigate the causes of the epidemic, thinks that it has as yet, by no means, reached its culmination, 50 per cent. of the cases not having arrived at the critical stage. He attributes it, in large part at least, to the suspension of the mechanical filtration of the city water supply. Unfiltered water from Conquonessing Creek was supplied from October 26 to November 2, the epidemic beginning November 5, 1903. Dr. Soper states that investigation of the drainage areas showed the existence of numerous sources of pollution and particularly the occurrence of cases of typhoid on the banks of the Conquonessing since July.

It will be remembered that a similar epidemic occurred at Plymouth, a mining town near Wilkesbarre, Pa., in 1885, which was appalling in its devastation, over 1,100 cases having developed in a population of 8,000. Last year at Ithaca an outbreak of the disease among a population similar in size to Butler, caused about 900 cases of typhoid fever. And yet the disease in question belongs to the preventable class! A grave responsibility rests upon public health officials everywhere throughout the land. Their inefficiency is at once demonstrated wherever such an outbreak occurs.

---

APATHY regarding the value of research in the domain of medical science is one of the regretful features of the American temperament, individual and governmental. It is quite true that a number of wealthy men, of which Mr. Rockefeller and Mr. Carnegie may be mentioned as a type, have given liberally for the establishment and endowment of research laboratories. But these instances are exceptional. The great mass of our people pay little heed to this important topic and the government does almost nothing, comparatively speaking, to foster it.

We are led to these observations from the remarks made by Dr. Roswell Park at the Liberal Club dinner, December 18, 1903, during the course of which he reminded his audience that the city of Buffalo has a university with nearly a thousand students in attendance; that in its several departments it has done admirable work for many years, in proof of which he pointed to the fact that hundreds of its graduates have won distinction in divers ways; and yet, he affirmed, it has never had financial support from the city. It has a laboratory of research, he continued, that is engaged in a field of great concern to the health of humanity,—a cancer laboratory that not only fails to receive support from the city itself, but is compelled every winter to put up a desperate fight in the legislature for the comparatively trifling sum appropriated by the state for its maintenance.

"We need an awakening," said Dr. Park, "in our own city to the value of research, its marvelous benefit to the public, and also to its costliness and the great difficulty under which it is carried on."

This is a fact that cannot be denied, and it would seem high time for our citizens to arouse themselves from the lethargy that appears to have overcome them in this respect.

---

A JOINT committee representing the American Medical and American Pharmaceutical Associations has been appointed to investigate the desirability of establishing a National Bureau of Medicines and Foods. The committee requests publication of this fact and also that opinions, criticisms and suggestions on the subject be forwarded to the committee, the personnel of which is as follows: on the part of the American Medical Association, E. Eliot Harris, New York; N. S. Davis, Jr., Chicago; Solomon Solis Cohen, Philadelphia; H. Bert. Ellis, Los Angeles; Philip Mills Jones, San Francisco; on the part of the American Pharmaceutical Association, H. H. Rusby, New York; Jas. M. Good, Saint Louis; C. S. N. Hallberg, Chicago; A. B. Lyons, Detroit; S. A. D. Sheppard, Boston. Chairman, joint committee, H. H. Rusby; secretary, Philip Mills Jones.

---

EXPLANATION.—The paper by Dr. D. W. Cathell, entitled, Was it wise for the American Medical Association to change its code of ethics? was read at the meeting of the Medical and Chirurgical Faculty of the State of Maryland, September 24, 1903, and was printed in American Medicine, October 17, 1903. The author's reprint used for "copy" was made to state these facts, but the printer of this journal failed to use them, which we regret.

